# Blood Pressure Reactivity to an Anger Provocation Interview Does Not Predict Incident Cardiovascular Disease Events: The Canadian Nova Scotia Health Survey (NSHS95) Prospective Population Study

**DOI:** 10.1155/2012/658128

**Published:** 2012-02-08

**Authors:** Jonathan A. Shaffer, Lauren Taggart Wasson, Karina W. Davidson, Joseph E. Schwartz, Susan Kirkland, Daichi Shimbo

**Affiliations:** ^1^Center for Behavioral Cardiovascular Health, Department of Medicine, Columbia University Medical Center, New York, NY 10032, USA; ^2^Department of Psychiatry, Stony Brook University, Stony Brook, NY 11794, USA; ^3^Department of Community Health and Epidemiology, Dalhousie University, Halifax, NS, Canada B3H 3J5

## Abstract

We examined the association between blood pressure (BP) reactivity to an anger provocation interview and 10-year incident CVD events in 1,470 adults from the population-based 1995 Nova Scotia Health Survey (NSHS95). In an unadjusted model, those in the highest decile of systolic BP reactivity were more than twice as likely to have an incident CVD event compared to those in the decile with no reactivity (HR = 2.33, 95% CI = 1.15 – 4.69, *P* = 0.02). However, after adjusting for age and sex, and then also for Framingham risk score, body mass index, and education, this relationship was attenuated and not statistically significant. Diastolic BP reactivity was not associated with CVD incidence in any model. Individual differences in BP reactivity to a laboratory-induced, structured anger provocation interview may not play a major role in clinical CVD endpoints.

## 1. Introduction

A longstanding hypothesis in the field of behavioral medicine holds that individuals who are prone to experiencing large increases in blood pressure (BP) during psychological stress are at increased risk for developing preclinical and clinical cardiovascular disease (CVD) states [1]. Indeed, evidence from prospective studies of the association of stress-associated BP reactivity with preclinical disease, such as atherosclerosis or left ventricular hypertrophy, is fairly consistent [2]. A recent meta-analysis showed that greater cardiovascular responses (systolic or diastolic BP) to laboratory-based psychological stress tasks are associated with greater risk of incident hypertension and progression of carotid intima-media thickening [3]. Individual studies have likewise found that excessive BP responses to the threat of shock or to a cold pressor task predict 10-year elevated blood pressure among initially normotensive young men with a family history of hypertension [4]. Moreover, BP reactivity to psychological stress has been shown to predict incident hypertension at 4-year, 10-year, and 13-year follow-ups among 508 normotensive Finnish middle-aged men [5], 796 English male civil servants [6], and more than 4,100 multiethnic participants in the Coronary Artery Risk Development in Young Adults (CARDIA) study [7], respectively. Elevated BP reactivity to stress has also been linked to carotid intima-media thickness in middle-aged Finnish men [8–10], progression of carotid atherosclerosis among non-medicated patients with existing atherosclerotic disease [11], and coronary artery calcification among a cohort of healthy young adults [12]. 

These preclinical disease states are notable health outcomes, but it is essential to consider whether BP reactivity to psychological stress ultimately predicts CVD events. Longitudinal studies of the association between BP reactivity and clinical endpoints, which are scarce, have shown mixed results [2]. For example, the findings of one study that linked stress-related increases in diastolic BP to myocardial infarction or cardiac mortality [13] were not replicated in either of two subsequent studies [14, 15]. The interpretation of these equivocal results is complicated by methodological issues, such as differing assessments and definitions of BP reactivity, a lack of time-to-event data in some studies, failure to control for traditional CVD risk factors that may confound the BP reactivity-CVD association, and a reliance on the cold pressor task as a psychological stressor rather than on tasks that are analogues of real-world situations such as anger recall and structured interviews. Further, participants were typically not from epidemiologic samples, limiting the generalizability of the results to the general population. 

In this study, we examined the association between BP reactivity to a structured anger provocation interview and 10-year incident CVD events in a population-based sample of 1.470 CVD-free individuals from Nova Scotia, Canada.

## 2. Materials and Methods

### 2.1. Participants

The 1995 Nova Scotia Health Survey (NSHS95) is a population-based survey implemented by Heart Health Nova Scotia and the Nova Scotia Department of Health to estimate the distributions of selected health indicators and preventive practices of Nova Scotians [16]. Participants were selected based on a probability sample designed by Statistics Canada, the national statistical agency and census bureau, and are representative of the Nova Scotian population in terms of age, sex, and geographic location. Study participants were non-institutionalized Nova Scotians, 18 years of age or older, and listed in the Medical Services Insurance registry, the government-sponsored universal health insurance plan. Pregnant women were excluded from the survey. The overall recruitment percentage (72%) is comparable to those reported in other large health surveys, with a final survey sample size of 3,227 participants. Propensity score analyses revealed no meaningful response biases [17]. Although demographic shifts have occurred in Nova Scotia, including a decreased rate of population growth, greater immigration than emigration, and an increasing median age, [18] we have no reason to suspect that these changes would alter the results of the analyses reported below.

We restricted our analysis to participants who had attended the clinic session and those without hospital discharge diagnoses of CVD during the 5 years before the baseline survey, as determined by International Classification of Diseases, Ninth Revision (ICD-9) [19] codes 410.X through 414.X (ischemic heart disease), 443.X (peripheral vascular disease), and 430.X through 435.X (cerebrovascular disease). Survey respondents were excluded because of refusal to permit linkage to medical outcomes (*n *= 312), preexisting CVD (*n *= 451), failure to attend the clinic visit (*n *= 388), failure to complete the Expanded Structured (anger provocation) Interview (*n *= 391), or lack of a resting BP or BP reactivity assessment (*n *= 215). Eight additional participants did not have data on diastolic BP reactivity, leaving 1,470 participants for the primary analysis of systolic BP reactivity (746 males, 724 females), and 1,462 participants for the secondary analysis of diastolic BP reactivity (743 males, 719 females). Participants who were excluded from our analyses had significantly higher levels of HDL cholesterol and were more likely to be female, a current smoker, and not have a high school education compared to those who were included.

### 2.2. Study Design

A group of 29 registered public health nurses were trained in standardized data collection and contacted potential survey participants by telephone from March through November 1995. Consenting participants were interviewed at home and then seen in clinic, approximately one week later, for measurement of height and weight and to provide a fasting blood sample. During the clinic assessment, participants completed a videotaped structured anger provocation interview that was subsequently reviewed and scored. Participants provided consent to store and use videotapes and to link future ischemic heart disease events with prior health care utilization. Additional study details are reported elsewhere [20, 21]. This study was approved by the Institutional Review Boards of Dalhousie University, Halifax, Nova Scotia, and Columbia University, New York, NY.

At baseline, each component of the Framingham risk score [22] was assessed, including sex, age, total and high-density lipoprotein (HDL) cholesterol levels, blood pressure, history of diabetes, and cigarette smoking. Total and HDL cholesterol levels were assayed from plasma samples by the Lipid Research Laboratory, University of Toronto, Toronto, Ontario [23]. Registered nurses used manual sphygmomanometers to measure resting systolic and diastolic BP. Two readings were obtained in the participant's home, two readings were obtained during the clinic visit (approximately 1 week later), and the average of these four readings was computed as a measure of resting systolic and diastolic BP. History of diabetes and completion of a high school education (“yes” versus “no”) were ascertained by self-report. As per the Framingham risk score calculation [22], those who reported smoking currently or in the past year were categorized as smokers; all others were categorized as nonsmokers. Weight and height were measured twice, averaged, and used to calculate body mass index (BMI; calculated as weight in kilograms divided by height in meters squared). Hypertension was defined as current use of antihypertensive medication or resting systolic BP ≥ 140 or diastolic BP ≥ 90 (using the aforementioned measure of resting BP obtained during home and clinic visits).

The Expanded Structured Interview (ESI) is a 12-minute, interpersonally stressful interview designed to elicit anger and stress by asking participants about their characteristic responses to a variety of different situations (e.g., performing a task at work while under pressure, waiting in traffic, and playing a competitive game) [24]. The interview is based on the original Type A Structured Interview [25], with additional questions on anger expression and interpersonal stress at work. Recent analyses of the NSHS95 data document that a substantial majority (89.9%) of participants who completed the ESI was judged by trained observers as having displayed at least some hostility, either in the content of their responses or the tone of their speech [26]. Nurse interviewers measured BP just prior to the ESI, at the midpoint of the interview, and at the end of the interview with a manual sphygmomanometer. Given that the ESI is designed to elicit maximal anger at its midpoint, the second of the three interview-based BP measures described above was selected as the measure of peak reactive BP. Systolic and diastolic BP reactivity were defined by subtracting resting BP from the second of the three interview-based BP assessments (i.e., a change score was computed). 

Nurse interviewers were trained on how to properly conduct the ESI during a four-part training procedure: (1) an ESI workshop, (2) ESI practice, (3) feedback after reviewing the ESI practice tapes, and (4) feedback during the NSHS95 study. The ESI administration training workshop used methods that were similar to the training used by the CARDIA investigation, in which investigators were taught voice emphasis, question pacing, interview length, general question and probe content, and demeanor. After the training workshop, nurses completed 10 practice ESIs that were rated on five structured interview quality control measures (elaborations, empathy, length, presentation, and overlap). Written and oral feedback was provided to each ESI interviewer after the practice ESIs, and nurses began data collection only when their ESI interviewing skills were considered acceptable. During NSHS95 data collection, a random 5% of each nurse interviewer's ESIs were coded for the ESI quality control measures. Oral feedback about the quality control results was provided periodically throughout data collection to ensure that all interviewers maintained adequate ESI administration competence. 

The main study outcome measure was the time-to-first-event defined as incident fatal or nonfatal CVD as determined by the ICD hospital discharge codes defined above and/or by death certificates. Events data were gathered from the provincial health care database through March 31, 2005, the 10-year period after the initial enrollment of subjects. Given that Nova Scotia provides universal health care insurance, and events occurring outside of Nova Scotia are reimbursed and captured, the accuracy of this measure of outcome assessment is high [27]. In the Canadian single-payer health system, physicians indicate underlying and contributing causes of death, which are subsequently recorded and submitted by nosologists as ICD codes upon death or discharge. A data quality committee from the Nova Scotia Department of Health meets with health records personnel to ensure accuracy, to conduct random chart reviews, and to adjudicate discrepancies in data entry. All deaths are reported to provincial offices and subsequently to the national census bureau (Statistics Canada), which applies a nationally consistent process of determining the underlying cause of death. Specifically, these data were converted to the ICD-9 codes by staff at Statistics Canada; and only those codes listed above (or the equivalent International Statistical Classification of Diseases, 10th revision (ICD-10) [28] codes) qualified as fatal CVD. Data were extracted by the Population Health Unit of Dalhousie University.

### 2.3. Statistical Analyses

Statistical analyses were performed using SPSS 18.0 [29]. When a limited number of items were missing for the Framingham risk score, we used a previously published regression-based approach to determine the best linear-predicted score based on the non-missing items [30–32]. For a given combination of missing items, scores were imputed only if the imputation equation predicting the total Framingham risk score from the non-missing items had an *R* ^2^ ≥ 75%; this condition was satisfied when data were available for at least five items from the Framingham risk score. The cohort was divided into deciles on the basis of the distribution of systolic BP reactivity (primary measure of reactivity) and diastolic BP reactivity (secondary measure of reactivity). Participants' baseline characteristics and their correlations with deciles of systolic and diastolic BP reactivity were examined using zero-order Pearson correlation coefficients for continuous variables and point-biserial correlation coefficients for binary variables. 

For the analysis of the association between BP reactivity and incident CVD events, Cox proportional hazards regression analyses were used to calculate the unadjusted and adjusted hazard ratios and 95% confidence intervals (CI) of CVD associated with deciles of systolic BP reactivity, controlling for age and sex in one model and additionally controlling for Framingham risk score, BMI, and high school education in a second model. The fifth decile, which contained individuals with zero systolic BP reactivity, was selected as the referent group for the systolic BP reactivity analyses. The fourth decile, which contained individuals with zero diastolic BP reactivity, was selected as the referent group in the diastolic BP reactivity analyses. The chi-square statistic (*χ* ^2^) was used to test whether the risk of incident CVD events varied by decile of systolic BP reactivity. All analyses were repeated using diastolic BP reactivity as a secondary measure of BP reactivity.

Additional analyses considered BP reactivity as a continuous variable and also examined possible interactions of BP reactivity (top 3 deciles (30%) versus middle 4 deciles (40%) and bottom 3 deciles (30%) versus middle 4 deciles (40%)) with traditional CVD risk factors, including age (median split), sex, baseline hypertension status, quartile of Framingham risk score, being overweight or obese based on BMI, and resting BP. Finally, to determine the robustness of the primary analyses, we conducted three additional sensitivity analyses using alternate definitions of BP reactivity and resting BP. In the first, resting BP was included as a covariate in all models given that some researchers have recommended the use of residualized change as a measure of reactivity rather than absolute change [33]. In the second, BP reactivity was defined as the average of the second and third BP readings taken during the structured anger provocation interview relative to resting BP, as averaging may enhance the reliability of this measure of reactivity [34]. In the third, resting BP was defined as the average of clinic BP readings with the exclusion of the BP readings obtained at home. The results for all three sensitivity analyses were similar to the results obtained from the primary analyses. As such, we do not herein report further on these analyses, as the results do not alter the findings of the study.

## 3. Results and Discussion

### 3.1. Results

Baseline characteristics of the 1,470 NSHS95 participants and their correlations with deciles of systolic and diastolic reactivity are reported in Table 1. The mean BP reactivity was 1.12 ± 11.04 mm Hg for systolic BP reactivity (range, −39–48 mm Hg) and 2.21 ± 7.36 mm Hg for diastolic BP reactivity (range, −22–27 mm Hg). Mean age and Framingham risk score were both positively correlated with deciles of systolic BP reactivity. Current smoking status, prevalence of hypertension, and mean resting systolic and diastolic BP were negatively correlated with deciles of systolic BP reactivity. Prevalence of hypertension and mean systolic and diastolic BP were also negatively correlated with deciles of diastolic BP reactivity. 

**Figure 1:**
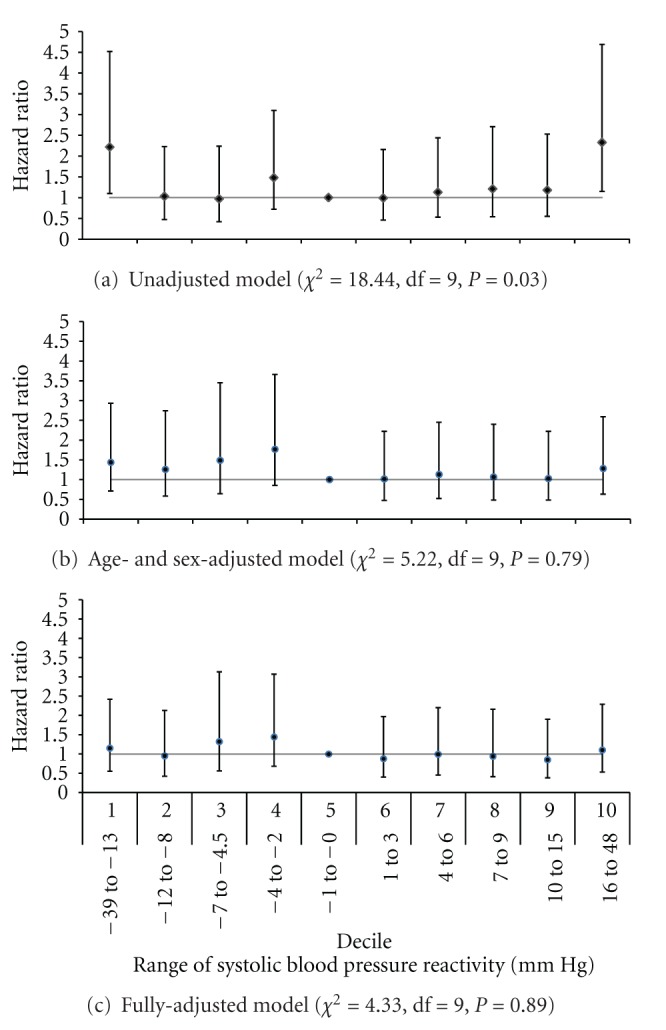
Hazard ratio with 95% confidence intervals of incident cardiovascular disease events by decile of systolic blood pressure reactivity in (a) an unadjusted model, (b) age- and sex-adjusted model, and (c) model additionally adjusted for body mass index, Framingham risk score, and high school education.

**Figure 2:**
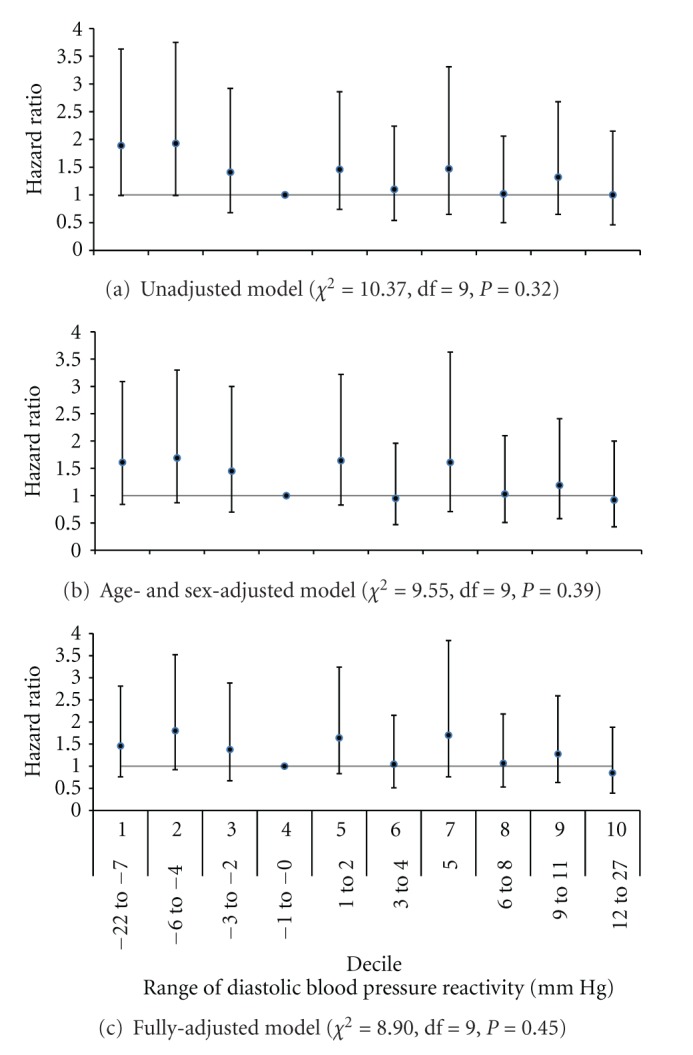
Hazard ratio with 95% confidence intervals of incident cardiovascular disease events by decile of diastolic blood pressure reactivity in (a) an unadjusted model, (b) age- and sex-adjusted model, and (c) model additionally adjusted for body mass index, Framingham risk score, and high school education.

A total of 161 nonfatal and 10 fatal incident CVD events occurred during the 10 years of follow-up for the 1,470 participants included in these analyses. In the unadjusted Cox proportional hazards regression model, systolic BP reactivity significantly predicted risk of incident CVD (*χ* ^2^ = 18.44, df = 9, *P* = 0.03) (Figure 1). Specifically, those in the lowest and highest deciles of systolic BP reactivity were, respectively, 2.2 (95% confidence interval (CI) = 1.10–4.53, *P *= 0.03) and 2.3 (95% CI = 1.15–4.69, *P *= 0.02) times as likely to develop incident CVD relative to those in the decile with no systolic BP reactivity. However, systolic BP reactivity no longer significantly predicted incident CVD events when age and sex were added to the model (*χ* ^2^ = 5.22, df = 9, *P *= 0.79) and it remained nonsignificant with further adjustment for Framingham risk score, BMI, and education level (*χ* ^2^ = 4.33, df = 9, *P *= 0.89). Results were similar when systolic BP reactivity was treated as a continuous rather than a categorical variable (data not shown). Diastolic reactivity did not predict incident CVD events in unadjusted (*χ* ^2^ = 10.37, df = 9, *P *= 0.32), age- and sex-adjusted (*χ* ^2^ = 9.55, df = 9, *P *= 0.39), or fully adjusted (*χ* ^2^ = 8.90, df = 9, *P *= 0.45) Cox regression models (Figure 2). 

We next considered whether exaggerated BP reactivity interacted with age, sex, hypertension status, being obese or overweight based on BMI, quartiles of Framingham risk score, or resting BP in the prediction of incident CVD. Neither systolic nor diastolic BP reactivity significantly interacted with any of these variables in the prediction of incident CVD (all *P*'s for interaction terms > 0.16).

### 3.2. Discussion

We evaluated whether BP reactivity to a structured anger provocation interview predicted incident CVD disease events in a prospective population-based study. In an unadjusted model, systolic BP reactivity to anger provocation significantly predicted risk of incident CVD such that those in the lowest and highest deciles of systolic BP reactivity were more than twice as likely to develop incident CVD compared to those in the decile with no reactivity. However, systolic BP reactivity, at any decile level, did not predict incident CVD events above and beyond traditional CVD risk factors, including age, sex, Framingham risk score, BMI, and completion of a high school education. Further, diastolic BP reactivity, at any decile level, did not predict CVD in unadjusted and adjusted models. These results are supported by a strong study design, including a large, population-based sample that was randomly selected, careful capture of CVD events at 10-year follow-up using hospital records, and the use of a psychological stressor that individuals will most likely experience in the natural environment. In addition, important potential confounders were considered, and analysis of possible interactions of BP reactivity with traditional CVD risk factors was conducted to elucidate whether certain subgroups were at risk. 

Research examining the relation between BP reactivity to a psychologically stressful interview and either preclinical or clinical CVD outcomes is limited. The single study that examined whether BP reactivity to a Type A structured interview predicts CVD events did not find a significant association in a small sample of participants with pre-existing CVD [35]. More recent evidence suggests that BP reactivity to being interviewed about a recent, stressful interpersonal situation predicts future BP [36]. Although this finding suggests that increased BP reactivity to a psychologically stressful interview may be associated with hypertension onset, there is little evidence that it is associated with clinically evident CVD.

 A few additional studies have examined prospective associations of BP reactivity to other psychological stressors of questionable ecological validity with CVD events. For example, one study reported a positive association of systolic BP reactivity with incident stroke [37] and another reported an association of diastolic BP reactivity with both myocardial infarction and total CVD [38]. However, the former study was limited to middle-aged men and the stressor was anticipation of an exercise test while the latter study enrolled only patients with hypertension, defined BP reactivity as the difference between nurse and physician assessments (a variant of the white-coat effect), and did not include any other type of psychological stressor. Other studies that have reported significant associations of BP reactivity with CVD events [39, 40] have been limited to patients with preexisting CVD, included relatively small samples of fewer than 100 participants, have not controlled for age, sex, and other traditional CVD risk factors, and/or have relied on cognitive tasks such as mental arithmetic and the Stroop test that likely have less ecological validity than the anger provocation interview used in the current study. 

 Additional prospective studies of the possible cardiovascular effects of BP reactivity have defined reactivity in response to the cold pressor task, with some of these showing that reactivity does predict incident CVD events [13] and others failing to replicate this finding [14, 15]. Given the use of the cold pressor task in these studies, however, their comparability to the current study is unknown. We elicited BP reactivity to a psychological stressor that is a laboratory analogue of a real-world situation rather than relying on a mixed physical/psychological stressor such as the cold pressor task. Although few, if any, studies have tested whether BP reactivity to anger provocation is a more valid method of eliciting reactivity than the use of a cold pressor task, people are more likely to experience anger in their daily lives than they are to hold their hands in near-freezing water for several minutes. In addition, the cold pressor task risks producing primarily reflex hemodynamic changes [3]. As such, our study findings likely have greater lab-to-field generalizability, with respect to psychological stress, than those of studies that have used the cold pressor task.

Although systolic BP reactivity predicted 10-year incident CVD events in an unadjusted Cox proportional hazards regression model in the current study, this finding was no longer significant after simple adjustment for age and sex. In addition, age was significantly correlated with deciles of systolic BP reactivity in bivariate analyses, suggesting that age is an important confounder of the relation between stress-related systolic BP reactivity and incident CVD events. Indeed, previous studies have demonstrated significant associations of age with systolic BP reactivity [41–44], lending support to the idea that age confounds the association of systolic BP reactivity with incident CVD events. Moreover, although some studies have suggested that the predictive validity of BP reactivity may differ for men and women [3], the association between BP reactivity and incident CVD in the current study was not modified by gender as well as other important characteristics such as age, hypertension status, resting BP, Framingham risk score, or being overweight or obese. 

There are several possible limitations to our study. First, our primary measure of BP reactivity was defined in relation to a single BP reading during the structured anger provocation interview. Previous studies have shown that the aggregation of cardiovascular responses across multiple time points and multiple stressful situations improves the reliability and generalizability of such measures [34]. Although we replicated our null finding using a measure of reactivity based on the average of the second and third interview-based BP readings, the reliability of our reactivity measure would have been increased even further if we had additional measures of BP during the ESI. Second, although some studies suggest that cardiac reactivity to an anger provocation interview in the laboratory is associated with reactivity in real life [45], such evidence is limited [46]. Indeed, the manual ascertainment of blood pressure in the middle of the anger provocation interview may not be comparable to typical experiences elicited in the natural environment. Third, this study did not include ascertainment of incident hypertension diagnosis or other preclinical disease endpoints as outcomes. Although future studies could examine whether reactivity to anger provocation is associated with hypertension and other preclinical outcomes, additional studies such as these may not be needed if BP reactivity does not ultimately lead to incident CVD events. Finally, the mean age of our sample was relatively young. Although we found that age of the participant at baseline did not interact with the relation between BP reactivity and incident CVD events, it is unclear whether our results can be extended to a population of elderly participants without initial CVD.

## 4. Conclusion

Neither systolic nor diastolic BP reactivity to anger provocation significantly predicted incident CVD events independent of traditional CVD risk factors, including age, sex, Framingham risk score, BMI, and education in a population-based study. These results suggest that BP reactivity to a laboratory-induced structured anger provocation interview does not play a major role in the development of CVD.

## Figures and Tables

**Table 1:** Baseline characteristics of 1,470 NSHS95 participants and their correlation with systolic and diastolic blood pressure reactivity to an anger provocation interview.

Characteristic	Total (*N* = 1,470)	Correlation* with deciles of systolic BP reactivity	Correlation* with deciles of diastolic BP reactivity
Mean age (SD), years	45.31 (17.9)	0.15^‡^	−0.01
Mean Framingham risk score (SD)	1.13 (9.25)	0.08^†^	−0.04
Mean BMI (SD), kg/m^2^	27.03 (5.39)	−0.03	−0.05
Mean LDL cholesterol (SD), mmol/L	3.22 (0.90)	−0.002	−0.001
Mean HDL cholesterol (SD), mmol/ L	1.26 (0.34)	0.02	0.03
Mean total cholesterol (SD), mmol/ L	5.28 (1.08)	0.01	−0.02
Mean resting systolic BP (SD), mm Hg	124.39 (18.65)	−0.14^‡^	−0.09^†^
Mean resting diastolic BP (SD), mm Hg	77.02 (9.54)	−0.08^†^	−0.24^‡^
Female, *n *(%)	724 (49.3)	−0.01	−0.05
High school education, *n *(%)	1,054 (71.8)	0.01	0.01
Current smoker, *n *(%)	388 (26.4)	−0.09^†^	−0.03
Hypertension, *n *(%)	375 (25.5)	−0.07^‡^	−0.13^‡^
Diabetes mellitus, *n *(%)	56 (3.8)	−0.01	−0.02

Abbreviations: BP, blood pressure; BMI, body mass index; LDL, low-density lipoprotein; HDL, high-density lipoprotein. *Associations of continuous variables with deciles of SBP and DBP reactivity are represented by zero-order Pearson correlation coefficients; associations of binary variables with deciles of reactivity are represented by point-biserial correlation coefficients. ^†^ *P* < 0.01; ^‡^ *P* < 0.001.
